# Artificial Intelligence as an Assistive Tool in Breast Cancer Screening and Diagnosis in the Digital Era

**DOI:** 10.1155/ijbc/5575969

**Published:** 2026-09-12

**Authors:** Manjit Kaur Rana, Amanjot Singh, Amritpal Singh Rana, Sukhchain Kaur, Aklank Jain, Astha Gupta, Upneet Kaur

**Affiliations:** ^1^ Department of Pathology, All India Institute of Medical Sciences Bathinda, Bathinda, Punjab, India; ^2^ Department of General Surgery, All India Institute of Medical Sciences Bathinda, Bathinda, Punjab, India; ^3^ Department of Zoology, Central University of Punjab, Bathinda, Punjab, India, cup.ac.in

**Keywords:** AI software validation, cancer screening, HER2 scoring, histopathology, tumor segmentation

## Abstract

**Objective:**

Breast cancer (BC) is a worldwide health task, and its increasing frequency and the associated burden on healthcare professionals are also increasing. Hence, integrating machine learning (ML) and artificial intelligence (AI) into screening and diagnosis is irrefutable in the digital age. A comprehensive systematic review was conducted aimed at assessing the application of AI and ML techniques to enhance BC screening, diagnosis, classification, and tumor marker scoring.

**Methods:**

An electronic literature search in PubMed and google scholar by following systematic review guidelines was conducted using keywords AI, cytology, histology, tumor marker expression, deep learning (DL) and ML and carcinoma breast. Articles exclusively pertaining to BC pathology were included in the study.

**Results:**

This study reviews the AI tools incorporating advanced DL models, such as artificial neural networks (ANNs), support vector machines (SVMs), convolutional neural networks (CNNs), and faster R‐CNN, which have demonstrated high accuracy in analyzing medical images from various modalities, including cytology and histopathology. AI applications also extend to combined imaging‐pathology diagnostics and tumor biomarkers, showing promising outcomes for consistency, rapidity, and cost‐effectiveness. Various Food and Drug Administration (FDA) and internationally approved AI software products for BC detection illustrate the translation potential of these technologies.

**Conclusions:**

The use of AI in the analysis of pathological samples in diagnosis, research, precision medicine, and patient care is increasing, and such assistance has great potential to reduce the workload of pathologists.

## 1. Introduction

The Global Cancer Observatory estimated 19.3 million cases of cancer worldwide, attributed to the elevated incidence of cancer patients worldwide [1]. The World Health Organization′s (WHO) Global Cancer Observatory (GLOBOCAN) documented 1,413,316 (male: 691,178 and female: 722,138) new cases in the 2022 statistical fact sheet. The researcher also depicted that the burden will rise to 57.5% in two decades (2020–2040). In India, lung and breast are leading carcinomas with breast cancer (BC) being the most common carcinoma in females [2]. Fine‐needle aspiration cytology (FNAC) and core needle biopsy (CNB) are two suitable assessment criteria used by pathologists. Due to rising workloads, the complexity of precision pathology and interobserver variability, traditional methods of interpretation of histopathology are becoming more and more challenging. With recent developments in modern technology, digital pathology and AI could contribute to the accurate and reliable diagnosis of carcinoma [3]. In the current review, an assessment of published literature for articles in Google Scholar and PubMed databases was conducted to illustrate how AI technologies and machine learning in the digital age are being used to empower healthcare professionals and assist in the screening and diagnosis of BC.

## 2. Method

A systematic literature search was made in English using electronic databases. The updated “Preferred Reporting Items for Systematic Reviews and Meta‐Analyses (PRISMA)” guidelines were followed. A literature review was systematically done for research papers by using keywords and MeSH terms like AI, cytology, CNN, histopathology, immunohistochemical (IHC), DL, and ML, and so on. A total of 122 original research and review articles were reviewed, and 58 were included in the study.

## 3. Results

The AI tools integrated with ML and DL are artificial neural networks (ANNs), support vector machines (SVMs), and CNNs with many branches. AI tools effective in cytology were deep neural networks (DNN), ML trained model, neuronal network technology, and feed‐forward neural network‐based (FFNN) with accuracy ranging from 89.3% to 98.6%. For histopathology of BC, various tools were validated for the detection of normal breast tissue, proliferative lesions, carcinoma in situ, and invasive carcinoma. In addition, tools have been manufactured to check the aggressiveness of tumors by evaluating grading, mitosis, and tumor marker status.

### 3.1. AI and BC Diagnosis

The diagnosis of BC has two important aspects: one is imaging of the tissue, and the other is the pathological sample taken from cells or tumor tissue, in addition to clinical examination by a surgeon. Imaging is performed by using various imaging techniques, including sonography, magnetic resonance imaging (MRI), x‐ray, and mammography, which are compatible with AI assessment [1]. AI in breast pathology has been applied in various tasks to diagnose and classify breast lesions, histopathological grade, BC, and metastatic deposits. The manual assessments of tissue for routine diagnostic factors, hormone receptor, human epidermal growth factor receptor 2 (HER2) neu, and Ki 67 analysis are subject to the experience and judgment of the pathologist, and patients have to wait for the final reports. These days, research published by many authors (from computer, data science, and pathology) has illustrated that the assessment can be made more convenient, and reliable results can be drawn with the help of artificial intelligence [2]. The review analysis of histology image analysis to BC detection and grade was done by He et al. [4], including 161 studies favoring mean shift clustering, Markov random field‐based Bayesian segmentation models for histology image segmentation. In addition, several algorithms and assessment technologies have been tested, providing a range of classification accuracies with the accepted sensitivity. Computer‐aided detection/diagnosis (CADe/CADx) underlying DL algorithms was considered a new advancement in AI, due to improved neural network (NN) architectures biologically with subsequent accurate data‐driven conclusions. When compared with old ML techniques selecting features manually, DL implements backpropagation processes and permits computerized knowledge and optimization [3]. The most frequently used models in pathology image analysis under branches of DL are CNNs, in which beforehand trained filters are being used onto input images, resulting in the extraction of complex characteristics of tissue cells, ensuing in detailed detection of patterns [5]. With the help of whole slide image (WSI) scanners, digitization of glass slides converting into high‐resolution digital images is done under digital histopathology. The several algorithms and assessment technologies have been tested since then in AI, providing a range of classification accuracy with the accepted sensitivity.

### 3.2. AI and Cytopathology

The potential of digitization and AI in cytopathology is significant. The AI technology is capable of assisting and reducing the burden on pathologists and also supporting timely reporting, which eases the waiting time for patients. As cytopathological examination serves as a tool for diagnosing breast lesions, many researchers proposed several algorithms and demonstrated their effectiveness. Huge cytopathology data is being assessed to increase the accuracy, sensitivity, and specificity of WSI. ML and AI techniques have been progressively applied to automate and enhance diagnostic accuracy, especially for cytological samples, such as FNAC images. Various ML algorithms, including ANNs, SVMs, fuzzy extreme learning machines (ELMs), DNNs, and ensemble methods, have been evaluated on diverse datasets, such as the Wisconsin Breast Cancer Dataset (WBCD) and digital cytology images. Amongst all, DNNs have shown promising results. Karthik et al. [6] reported accuracies exceeding 98.6% using DNNs with recursive feature elimination on the WBCD dataset, outperforming traditional SVM‐based systems in classification tasks. Sadhukhan et al. [7] used an ML‐trained model working on the cell nuclear features. The classification applied to images was of three levels: black, white, and grey. A significantly high level of efficiency (97.49%) was observed, augmenting the use of AI in cytopathology of breast carcinoma. On further research in 2022, Amethiya et al. reviewed new technology of multiple ML algorithms combined with biosensors, which demonstrated the potential for rapid and sensitive detection using electrochemical and field‐effect transistor (FET)‐based biosensors. These biosensors complement ML approaches by offering additional modalities for early detection beyond image analysis [8].

In order to justify the utility of AI in conditions where expert cytopathologist is not easily available, Fritz et al. [9] performed study of context variables for testing the validity and likelihood of cytologic interpretation for image classification in case of cytological screening by inexperienced personnel. Application in clinical settings was explored by Azam and associates in 2024, and they observed 99.4% of clinical management concordance (CMC) in a study done on 608 BC cases along with cancer screening samples CMC rates of 96.2% [10]. In a recent study, Islam et al. [11] developed a FFNN system that analyzes nuclear features from FNAC samples, achieving an accuracy of 98.10% on WBCD, the results demonstrated the reliable differentiation between benign and malignant cytology samples. These studies illustrate the potential of advanced machine learning techniques and assistance of AI in BC cytological screening (Table 1).

**Table 1:** Data showing accuracy of AI tools in BC cytology.

Author name, year	Tool used	Accuracy
Karthik et al. 2018 [6]	DNN	98.62%
Sadhukhan et al. 2020 [7]	ML trained model	97.49%
Fritz et al. 2023 [9]	Neuronal network technology	89.3%
Islam et al. 2024 [11]	FFNN	98.10%

### 3.3. AI and Histopathology of BC

In the present scenario, not only primary screening on FNAC, AI algorithmic programs are also being drafted to support pathologists in the diagnosis, grading, and histopathological classification of BC. BC histopathology is the gold standard technique for tumor diagnosis. Digital pathology and computational image analysis of high‐quality digital images empower the precise histopathological analysis of histological complex patterns of tissue. Further, the application of AI in histological assessment shows more accurate and reproducible results in comparison to the manual method for histopathological examination. Though traditional histopathological assessment remains the gold standard method of diagnosis, the incorporation of AI‐based tools is gradually refining accuracy and reproducibility in histopathological assessment [12].

One decade ago, a visual semantic analysis (VSA) was done on 162 IDC cases by Cruz‐Roa A et al. [13] constituting 113 training and 49 independent testing cases. Upon evaluation, F‐measure scored was 71.8% and balanced accuracy was detected to be 84.2%. Simultaneously, Romo‐Bucheli et al. [14], worked on AI and histological grading of BC by considering tubular formation and nuclear characteristics. A DL‐based CNN classifier was engineered for automation in identifying nuclei of tubules from WSI. Subsequently, Oncotype DX risk categories were assessed and correlation area under the receiver operating characteristic curve (AUC) of approximately 0.76 was gained. Their work in application of AI in automatic nuclei segmentation of tubules provided revolutionary results by gaining an independent prognostic value (0.032). A DL model formulated by Han et al. [15], was validated on a huge dataset for diagnose and classification of BC with average accuracy of 93.2%. Furthermore, for use of AI in categorization of histological types, Yamamoto et al. [16] established a model by evaluating 11,661 myoepithelial cell nuclei. The unique flattened shape of myoepithelial nuclei cells was detectable by computational methods and was categorized into histological subcategories (four) with accuracy of 90.9%. Bejnordi et al. [17] framed more precise context‐aware stacked CNN and classify the breast WSIs into normal/benign, ductal carcinoma in situ (DCIS), and invasive ductal carcinoma (IDC). The model was evaluated on 221 WSIs of hematoxylin and eosin‐stained (H&E) breast tissue sections. A three‐class accuracy of 81.3% for all categories was demonstrated, highlighting the potential of model for routine diagnostics. Subsequently Cruz‐Roa et al. [18] studied histopathology image analysis (HASHI), a CNNs based powerful AI tool invented to determine IDC in a WSI by probability gradients and quasi‐Monte Carlo sampling method. HASHI was capable of assessing 2000 samples in just 1 min. Furthermore, Fondon et al. [19] developed a CAD tool‐based SVM classifiers with quadratic kernels. The model showed accuracy levels of 75.8%, 75%, and 61.11% in classifying breast samples into normal, benign, in situ, and invasive, respectively. Many researchers worked to improve the applicability of AI in breast carcinoma with accuracy ranging from normal breast tissue (75.8%–81.3%), proliferative lesions (85.7%), benign (75.8%–81.3%), DCIS (75.0%–96.0%), and IDC (61.5%–97.7%) (Table 2).

**Table 2:** Details of tools and BC histopathological tissue used and outcome of AI.

Author, year	AI tool	No. of images/samples analyzed	Type of tissue	Results
Cruz‐Roa A et al. 2014 [20]	VSA	162	IDC	F‐measure (71.8%) accuracy (84.2%)
Bejnordi et al. 2017 [17]	Context‐aware stacked CNN	221	Normal/benign, DCIS, and IDC	Accuracy (81.3%)
Cruz‐Roa et al. 2018 [18]	HASHI	2000 slides/min	IDC	Reduced time consumption
Fondon et al. 2018 [19]	CAD tool based SVM classifiers	—	Normal/benign, DCIS, and IDC	Accuracy (75.8%, 75%, and 61.11%)
Abdolahi et al. 2020 [21]	Pretrained VGG‐16 CNN model	277524 image patches	IDC	Accuracy (85%)
Barsha et al. 2021 [22]	Ensemble model	Large data set	IDC	Accuracy (92.70%) F1‐score (95.70%)
Voon et al. 2022 [23]	Seven selected CNN models with transfer learning	888 images	IDC grading	Accuracy for EfficientNetV2B0‐21 k (93.6%)
Celik et al. 2020 [24]	Pretrained models	277,524 image patches	IDC	DenseNet‐161 (F‐score: 92.38%, accuracy: 91.57%) ResNet‐50 (F‐score: 94.11%, accuracy: 90.96%)
		BreakHis breast cancer dataset	Classification of IDC	Validated
Abdelli et al. 2020 [25]	Multi‐CNN architectures, ResNet50, and MobileNet	Lightweight and heavyweight architectures	Grades	High accuracy
Sato et al. 2021 [26]	Gradient‐weighted class activation mapping (Grad‐CAM)	—	Preneoplastic and neoplastic ductal lesions	AUC: 0.99–1.00
Kanavati et al. 2022 [27]	Trained DL models	1382, 548	DCIS, IDC, and benign	Accuracy (DCIS, 96.0%), (97.7% IDC)
De Assis et al. 2022 [28]	CNN for VSA	High volume data	IDC	F1‐score of 0.85
Islam et al. 2024 [29]	(CNN) model	277,524 total images	IDC and metastatic breast cancer	Accuracy (95% metastasis), (89% IDC)
Masood et al. 2026 [30]	Pathology Artificial Intelligence Guidance Engine Breast (Paige PaBr)	250	CNB	Sensitivity and specificity of 90.2%, and 89.5% (suspicious lesions) sensitivity (97.4% IDC) and (85.7% DCIS, proliferative lesions)

### 3.4. AI and Mitosis Evaluation of BC

The efforts were made to enable AI to assess mitosis to analyze tumor aggressiveness. In 2013, a deep max‐pooling CNN tool was used by Cireşan et al. and mitosis was recognized in breast histology images [31]. Other researchers like Cireşan et al. [31], Xu et al. [32], Dodballapur et al. [33], Tang et al. [34], and Li et al. [35] also validated AI tools to assess the aggressiveness of BC by analyzing mitosis in the tumor tissue. Veta et al. [36], emphasized that the Assessment of Mitosis Detection Algorithms 2013 (AMIDA13) had revealed an overall F‐1 score of 0.61 versus manual method by using a 10‐layer deep CNN algorithm with overall F‐1 score of > 0.75. Furthermore, Veta et al. [37], Tumour Proliferation Assessment Challenge 2016 (TUPAC16) was formulated for evaluation of mitotic scores on WSIs and F‐1 score of 0.65. However, detection of moderate F1‐scores in range of 0.65–0.75 highlights the possibility of heterogeneous datasets, biological variability in tumor and variations in annotation. Pantanowitz et al. [38] concluded that in evaluation of mitotic figures was more accurate when done by pathologist supported by AI. With AI assistance, false positives counts were reduced by 54.2%, detection was improved by 87.5% and time spent was decreased by 27.8%. Based on digital WSIs and DL, Wang et al. [39], successfully validated a novel DeepGrade (histological grade model) model. A review analysis on 93 studies done by CVL et al. in 2024 emphasized the integration of ML and DL algorithms and appreciated the favoring results in diagnosis of BC [40].

### 3.5. AI and IHC

IHC staining for BC tumor markers like ER, PR, HER2 neu and Ki 67 expression is used to identify histologic subtype or molecular phenotype and still modify the treatment plan for patient IHC. BC molecular subtypes are classified into four molecular types such as Luminal A, Luminal B, HER2‐enriched and basal‐like [41, 42]. The interpretation of tumor biomarkers shows observer variability and also is time consuming. The precision of the AI tools indicates that over time, the accuracy and consistency have increased [43]. The diagnostic accuracy also depends on various aspects, such as the standard used by the pathologists to determine HER2 scoring, type of staining, quality of imaging, and selection of a particular region of interest (ROI) or the whole slide, all of which influence the accuracy and consistency. Furthermore, quantitative biomarker evaluation can be benefited by digital image analysis (DIA), so quantitative image analysis (QAI) of 1367 BC cases was conducted. In addition, multiple ROI were selected by pathologist on particular area or on whole image slide of H&E‐stained tumor tissues. Concordance rate of 93%, 96%, and 90% was detected with manual assessment of ER, PR, and HER2, respectively [6, 44].

ER/PR‐positive BC responds to hormonal therapy and valid determination of expression is an utmost requirement for establishing treatment strategies [45]. The assessment of ER/PR expression is routinely done by Allred scoring by manual method [46]. Andersen et al. evaluated the value sensitive design (VDS) application for the finding the expression of ER in 2018. With the help of histoscore method an effective correlation between VDS and visual assessment (*R* = 0.950) was observed. Ultimately, image analysis system and human scoring for ER status was found to be with 1% cut‐off [47]. Ahmad Fauzi et al. [48] also anticipated a CNN model for Allred scoring system for hormone receptor status of BC.

A team of researchers, including pathologists, has illustrated that CNN‐based artificial intelligence can be applied to HER‐2 scoring. Wu et al. [49] conducted a two‐ring study with 15 pathologists from three different hospitals with different working experience (1–10 years) and performed HER‐2 neu interpretation. In ring one, the pathologists reviewed the slides and interpreted 246 IHC sections, whereas in ring two, AI was used on the same sections and slides. The study is aimed at developing a computer algorithm that is efficient in analyzing the images and subsequently quantifying the stains in the HER‐2 membrane; the results from this study demonstrated improvement in accuracy and consistency. This study used the HER‐2 neu scoring algorithm based on membrane delineation and cell classification. In particular, red, green, and blue (RGB) images were the inputs, and the output was a heat map. The accuracy and consistency of AI in evaluation of HER2 with IHC score 0 and 1+ in routine and in respect to tumor heterogeneity was done. The AI showed superior results in terms of precision for HER‐2 neu score 0 and improved the uniformity in the form of intraclass correlation coefficient (0.542–0.812) in HER2 1+ cases. On the other hand, cases of tumor heterogeneity also showed significantly improved accuracy from 0.68 to 0.89. The limitation is the selection of the field of view by AI assistance, and whole slide evaluation is subject to the will of the pathologist to examine. AI cannot differentiate between in situ and invasive carcinomas. Vandenberghe et al. [50] studied 335 digital microscopy images of HER2 neu using Aperio Scanscope T2 WSI, resulting in concordance of 83%. With more advancement, a team of researchers demonstrated a moderate agreement between DIA and app‐assisted reading (AR) for heterogeneous and irregular staining, with the use of HER2 CONNECT APP, an algorithm designed to measure the membrane staining in the region of interest. They also illustrated that DIA performed better and was helpful in the BC categorization of HER2 negative and HER2–0/+1 as compared with AR. However, the results from the study also found that for the categorization of HER2–0 and HER2–2+, AR performed better. Moreover, staining intensity ensures more accuracy and consistency [51]. Interpathologist variability is a subjective aspect with manual assessment for the interpretation of BC for HER2. With the advancement of AI, the DIA offers a potential solution to reduce interpathologist and intrapathologist variability. Researchers compared the concordance between the AI‐assessed HER2 status and the assessment performed by a pathologist. In a study, Palm et al. [52] demonstrated that the staining protocol recommended by the AI software was vital for diagnostic accuracy. With the application of recommended staining protocol, over two‐thirds of the cases (77.6%) showed concordance, which was 38.3% earlier. Recently, Dunenova et al. [53] reviewed 25 publications on DIA of HER2 images showing data of 84.19%–97.9% range in accuracy, reflecting the refining of AI techniques with time. However, no direct evidence supporting the clinical application was retrieved.

Liu et al. [54] analyzed the lymph node tissue data of 399 cases from the publicly available Camelyon16 challenge dataset. They observed the AUC of 99% and tumor sensitivity of 91% in lymphnode metastatic deposits. Although exploration of sentinel lymphnode biopsy (SLNs) was also done for AI application and clinical utility; however no promising results were perceived (Table 3).

**Table 3:** details the use of AI in SLN status detection.

Author, year	Tool	Samples	Result
Challa et al. 2023 [55]	Visiopharm Integrator System (VIS) metastasis AI algorithm	234 non‐SLNs,	Sensitivity/specificity (100%/78.5%)
		102 SLNs	Sensitivity/specificity (100%/41.5%)
Retamero et al. 2024 [56]	AI algorithm	≥ 32 000 breast SLN WSIs	Accuracy of 74.5%–93.5%

Andersen et al. [57]demonstrated the long‐term outcomes and cost‐effectiveness of Saige‐DX, an AI product cleared by the FDA for digital breast tomosynthesis screening, finding modest mortality reductions but concluding that current pricing is not cost‐effective. Allahqoli et al. [58]emphasized that ML can assess the risk and help in early detection of BC. However, large population‐based studies or large data set studies with clinical validation are the key of choice.

## 4. Conclusion

AI enhances the accuracy of pathology‐based BC diagnoses and therapeutic management. Digital pathology empowered by AI offers a solution to interpathologist variability in assessments, such as HER2 scoring. CNN‐based algorithms analyze stained tissue slides to quantify features, achieving precision and consistency comparable to or superior to manual evaluation. Techniques such as hybrid neural networks, genetic algorithms, and SVMs have achieved classification accuracies exceeding 95% across multiple datasets, demonstrating the capability of AI to standardize and accelerate cancer diagnostics.

AI‐driven tools are revolutionizing oncology by enhancing cancer detection through pathology and molecular imaging. Their integration promises faster diagnoses, reduced human error, and improved clinical workflows. Continued development and validation of AI algorithms will be crucial in addressing diagnostic disparities and strengthening global cancer care infrastructure.

## Author Contributions

Amritpal Singh Rana and Manjit Kaur Rana contributed to the origin, design, and execution of the publication. Amanjot Singh, Sukhchain Kaur, and Aklank Jain contributed to the study of the results and writing of the manuscript. Astha Gupta and Upneet Kaur contributed to the analysis of results.

## Funding

No funding was received for this manuscript.

## Conflicts of Interest

The authors declare no conflicts of interest.

## Data Availability

The data that support the findings of this study are available from the corresponding author upon reasonable request.
